# Research on reliability mapping of 5G low orbit constellation network slice based on deep reinforcement learning

**DOI:** 10.1038/s41598-024-66188-6

**Published:** 2024-07-03

**Authors:** Yunjie Xiao, Nan Li, Jiangtao Yu, Baozhu Zhao, Dawei Chen, Zhengrong Wei

**Affiliations:** Information & Communication Company, SMEPC, Jingan, Shanghai 200072 China

**Keywords:** Deep reinforcement learning, Resource demand, Functional virtualization, 5G low orbit constellation, Network slicing, Reliability mapping, Engineering, Mathematics and computing

## Abstract

Reliability mapping of 5G low orbit constellation network slice is an important means to ensure link network communication. The problem of state space explosion is a typical problem. The deep reinforcement learning method is introduced. Under the 5G low orbit constellation integrated network architecture based on software definition network (SDN) and network function virtualization (NFV), the resource requirements and resource constraints of the virtual network function (VNF) are comprehensively considered to build the 5G low orbit constellation network slice reliability mapping model, and the reliability mapping model parameters are trained and learned by using deep reinforcement learning, solve the problem of state space explosion in the reliability mapping process of 5G low orbit constellation network slices. In addition, node backup and link backup strategies based on importance are adopted to solve the problem that VNF/link reliability is difficult to meet in the reliability mapping process of 5G low orbit constellation network slice. The experimental results show that this method improves the network throughput, packet loss rate and intra slice traffic of 5G low orbit constellation, and can completely repair network faults within 0.3 s; For different number of 5G low orbit constellation network slicing requests, the reliability of this method remains above 98%; For SFC with different lengths, the average network delay of this method is less than 0.15 s.

## Introduction

Low orbit constellation network consists of a group of low orbit constellation satellites, forming a communication network covering the whole world or specific regions^1^. The maneuverability of LEO constellation network is fully reflected in the modeling through the rapid movement of satellites in LEO and the dynamic changes of network topology, as well as the resulting link stability, transmission delay and packet loss rate fluctuations. This mobility not only challenges the performance and reliability of network slicing, but also provides an opportunity for deep reinforcement learning algorithm to optimize network resource allocation and routing strategy, so as to achieve more stable and efficient network slicing service. Network slicing is a network virtualization technology, which can divide a physical network into multiple virtual networks, and each virtual network can run and manage independently^2^. 5G low orbit constellation network slicing is a network service mode combining 5G communication and low orbit constellation technology^3^. This network slicing technology can provide customized network services to meet the needs of different users^4^. Reliability mapping of 5G low orbit constellation network slice is an important means to evaluate and guarantee the reliability of 5G low orbit constellation network slice service^5^. Reliability mapping typically involves backup and recovery mechanisms for virtual network elements, such as nodes and links. Specifically, the technology aims to prevent and respond to virtual network failures to ensure the normal operation of business. In the network slicing environment, because the virtual network is built on the physical network, the reliability of the physical network has a direct impact on the reliability of the virtual network. Through reliability mapping, it can ensure that when the virtual network fails, it can quickly recover and continue to provide services. Due to the dynamic and high-risk nature of low orbit constellation satellites, the reliability of the network is facing great challenges.

For the reliability mapping method of 5G low orbit constellation network slice, some relevant experts and scholars have studied it. For example, Musumeci et al.^6^ proposed a double objective heuristic method. By simultaneously optimizing the two key objectives of the constellation network, namely, the number of active control terminals and the number of wavelength channels, the number of terminals is controlled by trying to minimize the required network resources, Minimizing the number of wavelengths required means optimizing the network capacity and improving efficiency. At the same time, this method optimizes under the constraints of network capacity and delay requirements to ensure that the generated slices meet the network capacity requirements, and the data transmission delay is within an acceptable range. However, this method may not guarantee to find a global optimal solution, but can only find a local optimal solution, In some cases, better solutions will be missed. Thiruvasagam et al.^7^ proposed reliability awareness, delay guarantee and efficient resource allocation methods for the slice service function chain in the software 5G network, proposed a new SFC subchannel method to improve the reliability of no backup SFC when mapping network slices, and converted the reliable SFC layout problem of network slice mapping into an integer linear programming (ILP) problem, However, the network slice mapping method has the problems of high computational complexity and long running time for large-scale input instances. Scancalepore et al.^8^ proposed a delay driven network slicing arrangement method in 5 g networks. Using a solid mathematical framework, the coordinator LACO based on multiple armed bandits (MAB) makes use of the exploration and development paradigm. The coordinator makes adaptive resource slicing decisions without prior knowledge of traffic demand or channel quality statistics, effectively managing and arranging network resources, To meet the needs of different users and businesses, LACO relies on system structure information to speed up decision-making. When LACO is in place, it measures the near optimal results within the affordable computing time, and dynamically adjusts according to real-time network conditions and needs, thus realizing the mapping of network slices. However, this method relies on real-time network status information. If the network status information is inaccurate or outdated, It may lead to mistakes in decision-making.

Deep reinforcement learning has adaptive decision-making ability^9^, which can enable agents to better cope with the dynamic changes and uncertainties in the low orbit constellation network. It is effective to optimize the allocation of network resources. Deep reinforcement learning Through learning historical data and real-time feedback^10^, agents can find the optimal way to use resources, thus improving the reliability of network slicing; Be able to process high-dimensional data, effectively extract useful features from a large amount of data, and provide more accurate prediction and decision support for reliability mapping; Deep reinforcement learning^11^ allows agents to accumulate experience through interaction with the environment and learn how to make optimal decisions, which can greatly reduce the need for manual intervention and make network management more automated and intelligent. Therefore, based on the shortcomings of the above methods, this paper studies the reliability mapping method of 5G low orbit constellation network slices based on deep reinforcement learning to improve the reliability and stability of network slices and meet the needs of users. Compared with traditional methods, the innovation of this method is:

1. The method of deep reinforcement learning is introduced. Under the integrated network architecture of 5G- LEO constellation based on software-defined network and virtualization of network functions, the reliability mapping model of 5G- LEO constellation network slices is constructed by comprehensively considering the resource requirements and resource constraints of virtual network functions, and the parameters of the reliability mapping model are trained and learned by deep reinforcement learning to solve the problem of state space explosion in the process of reliability mapping of 5G- LEO constellation network slices.
2. The node backup and link backup strategies based on importance are adopted to solve the problem that VNF/ link reliability is difficult to meet in the process of reliability mapping of 5G- LEO constellation network slices.

Compared with other methods, this method ensures the throughput, traffic transmission delay and data packet loss rate of 5G- LEO constellation network, and improves the load balance of 5G- LEO constellation network, thus improving the reliability of the network. Under the same number of requests, this method has higher reliability; According to the mapping of node reliability importance, resource allocation and redundancy management are carried out. This method can better identify key nodes and allocate more resources to them, thus improving the reliability and availability of network services.

## 5G low orbit constellation network slice reliability mapping

### Network architecture

**Figure 1:**
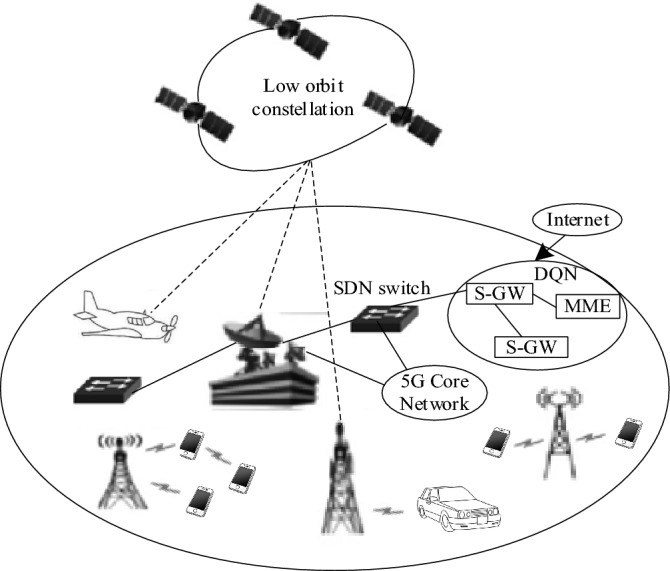
5G—Integrated Network Architecture of Low Earth Orbit Constellations.

The 5G low orbit constellation integrated network architecture is shown in Fig. 1^12^, which completes the organic combination of terminal equipment, satellite network functions, satellite baseband gateway (S-GW), mobility management entity (MME), core network resources and management system through software definition network (SDN) and network function virtualization (NFV) technologies, and divides the core network into core network processing cloud and core network forwarding cloud, Realize the separation of forwarding and control, so that it can provide maximum flexibility, openness and programmability. Among them, the mobility management entity MME in the network is an important network element, which is specially responsible for processing signaling, business control and user mobility management. It caches user information. Serving GateWay (S-G) plays a key role in request routing, security control, protocol conversion, etc. It provides a series of functions to ensure the reliability and security of the network.

Most functions of 5G low orbit constellation network slice are provided by the virtual network function (VNF) running in the distributed network function virtualization infrastructure^13^. In the ETSI ISG NFV terminology, the non virtualized functions of slices are expressed as physical network functions (PNF). Each 5G low orbit constellation network slice is composed of a series of VNF and PNF instances, which are linked together to form a service function chain (SFC) that lasts for a specific period of time. The service choreographer generates SFC according to service requests, uses mapping algorithms to realize network slice instantiation, and establishes a logically independent and mutually isolated network relying on satellite network infrastructure.

The problem of reinforcement learning is limited by multiple constraints, which include the challenge of data collection, the demand for real-time and dynamic, the balance between network performance and resource constraints, and the requirement of ensuring security and stability.

The service orchestration function of network architecture is usually deployed in the core part of the network, so that the network slicing resources can be managed, configured and optimized globally. In 5G and future network architecture, service orchestration can be deployed in multiple network elements, but it is most commonly deployed in central control nodes such as network management system (NMS), network function virtualization choreographer (NFVO) or network slice manager (NSM).

The deployment location of service orchestration function is as follows:

1. Network Management System (NMS): NMS is responsible for the management and monitoring of the whole network, including the management of network slices. Therefore, deploying the service orchestration function in NMS can ensure a global view of the whole network and directly manage the network slice resources.
2. Network Function Virtualization Orchestrator (NFVO): In NFV (Network Function Virtualization) architecture, NFVO is responsible for the life cycle management of network services, including instantiation, configuration, optimization and termination of network functions. Integrating the service orchestration function into NFVO can easily interact with NFV architecture and quickly respond to the needs of network slicing.
3. Network Slice Manager (NSM): NSM is a network element dedicated to managing network slice resources. Deploying the service orchestration function in NSM can ensure the direct control and management of slicing resources and improve the flexibility and efficiency of slicing configuration.

The deployment location of service orchestration function has an important influence on transmission delay. The qualitative analysis of transmission delay under different deployment locations is as follows:

1. Deployed in NMS: NMS is usually located at the core layer of the network and has good connections with other network elements in the network. Therefore, the transmission delay from NMS to each network slice is usually low. However, if the distance between NMS and the edge network nodes is far away, the management and response speed of edge slicing may decrease.
2. Deployed in NFVO: NFVO is usually located at the core layer of the network and closely integrated with virtualization infrastructure (such as VNFM). Therefore, the transmission delay from NFVO to virtualized network functions is usually low. However, if there is a bottleneck in the connection between NFVO and physical network nodes (such as base stations, switches, etc.), the management and response speed of physical network slices may decrease.
3. Deployed in NSM: NSM is a network element specially used to manage network slicing resources, and is usually closely connected with slicing related network elements (such as base stations, switches, etc.). Therefore, the transmission delay from NSM to each network slice is usually low, which can realize the rapid management and response of slice resources. However, if there are bottlenecks in the communication between NSM and NMS or between NMS and NMS and external systems (such as the coordination between NMS and NMS, the interaction between NMS and NMS and third-party systems, etc.), it may lead to the decrease of management and response speed across slices or systems.

### Network modeling

1. Physical networks.

The infrastructure layer provides the physical and virtual resources needed to create 5G low orbit constellation network slices^14^, and the underlying physical network is composed of weighted undirected graph $G_{s} = (N_{s} ,L_{s} )$, where $N_{s} = \left\{ {n_{1} ,n_{2} ,...,n_{M} } \right\}$ is the set of physical nodes, the $C_{i}$ denotes the computational power of the physical node $n_{i}$,$L_{s} = \left\{ {l_{ij} \left| {n_{i} } \right.,n_{j} \in N_{s} } \right\}$ denotes the set of physical node links, the $l_{ij}$ denotes specific physical nodes on the underlying physical network $n_{i}$, the physical link $n_{j}$, of which, the link failure rate of the communication link $l_{ij}$ is $\lambda_{ij}$, with a bandwidth of $B_{ij}^{s}$.

2. 5G low orbit constellation network slice request.

5G low orbit constellation network slice request $g$ is denoted by $G_{v}^{g} = (N_{v}^{g} ,E_{v}^{g} )$, where $E_{v}^{g} = \left\{ {e_{kg} \left| {v_{m} } \right.,v_{n} \in N_{v}^{g} } \right\}$ is a set of virtual links.$e_{kg}$ denotes the $k$ th virtual links of slice $g$.$C_{{s_{kg} }}$ and $C_{{d_{kg} }}$ denote respectively the computational resource requirements of the source and destination nodes of the $k$ th link of slice $g$.$N_{v}^{g} = \left\{ {v_{1}^{g} ,v_{2}^{g} ,...,v_{N}^{g} } \right\}$ is the set of virtual nodes, the $N$ denotes the number of virtual nodes of slice $g$, the $b_{g} = B(e_{kg} ),\forall e_{kg}$ denotes the required broadband capacity of slice $g$.

**Figure 2:**
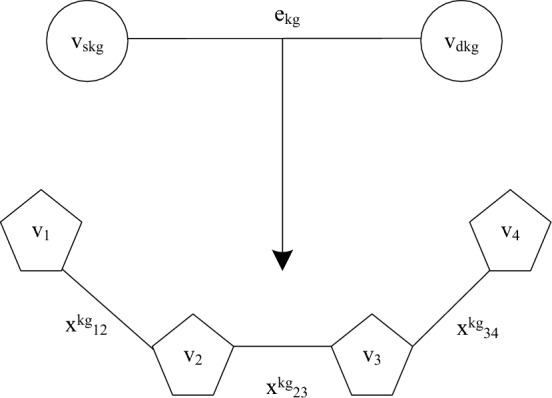
Virtual Link Two Point Subgraph Mapping.

As shown in Fig. 2, for each slice, mathematical models can be built using $K_{g}$ two-point directed subgraph representations, the $v_{{s_{kg} }}$ and $v_{{d_{kg} }}$ represent the source and destination nodes of the $k$ th virtual link $e_{kg}$, respectively, when the physical link $l_{ij}$ is selected, the $x_{ij}^{kg} = 1$, otherwise $x_{ij}^{kg} = 0$.

3. State, action and reward framework under deep reinforcement learning.
4. State:

State describes the complete situation of network slicing at a certain moment, including all kinds of observable and unobservable information.

In the context of reliability mapping of network slices in 5G- LEO constellation, the state includes the current load, delay, packet loss rate, bandwidth utilization rate and other performance indicators of network slices, as well as the position and orbit information of satellites and the connection state with other satellites or ground base stations.

The information integrity of state is very important for agents to make correct decisions. Therefore, when designing the state, it is necessary to ensure that the state can fully reflect the current situation of the network slice.

5. Action:

Action is an agent's behavior that is selected according to the current state and used to influence the environment.

In the context of reliability mapping of network slices in 5G- LEO constellation, actions include adjusting the resource allocation of network slices, changing routing strategies, optimizing transmission parameters, and so on. The purpose of these actions is to improve the reliability of network slicing, such as reducing delay and packet loss rate.

Action selection needs an agent-based strategy, which is usually trained by deep reinforcement learning algorithm.

6. Reward:

Reward is a feedback signal given by the environment after performing an action on an agent, which is used to guide the learning process of the agent.

In the context of reliability mapping of 5G- LEO constellation network slices, the reward can be defined based on the performance index of network slices. If the agent reduces the delay of network slicing or improves the bandwidth utilization through an action, it can give a positive reward; Conversely, if the performance drops, a negative reward will be given.

The setting of rewards has an important influence on the learning direction and speed of agents. Therefore, when designing the reward function, we need to ensure that it can accurately reflect the research objectives and optimization direction.

### 5G low orbit constellation network slice reliability mapping model

The mapping is to allocate the physical network resources to each VNF according to certain constraints. The service choreographer calculates the reliability requirements of a single VNF according to the reliability threshold of the service request. The physical nodes allocated by VNF need to meet the reliability requirements and computing resource constraints; The virtual link is mapped to a loop free path of the underlying physical network. The available bandwidth of each link on the path must meet the bandwidth requirements of the virtual link.

Network slice request (NSR) is a logical service formed by a group of VNFs through network interconnection^15^. According to VNF importance index, VNF reliability requirements are calculated to provide different reliability guarantees. When the overall reliability requirements of NSR $R_{req}^{g}$ is given, the RA assessment method can be used to obtain VNF reliability requirements. The three importance indicators defining VNF are: VNF $v_{i}$ computing resource requirements $S_{i} = C_{i}^{g}$; VNF bandwidth resource requirements, is defined as the sum $D_{i} = \sum\nolimits_{{ei,ei + 1 \in E_{g} }} {(b_{ei}^{g} + b_{ei + 1}^{g} )}$ of the bandwidth resources for all the adjacent links connected to the VNF $v_{i}$; VNF degree centrality, reflecting its importance from the perspective of VNF position, is defined as $M_{i} = 1/\sum\nolimits_{{v_{j} \in V_{g} }} {d_{ij} }$. Among them,$d_{ij}$ indicates the hop distance between VNF $v_{i}$ and $v_{j}$. The greater the centrality of VNF, the more important the VNF is in the middle of the network slice. Based on three importance indicators, the normalized weight indicator for $v_{i}$ is:

1 $$p_{i}^{g} = S_{i} + D_{i} + M_{i} /\sum\limits_{i = 1}^{{K_{g} }} {S_{i} + D_{i} + M_{i} }$$

According to the reliability calculation formula of components assessed by RA, it can be obtained that:

2 $$R_{i}^{g} = (R_{req}^{g} )^{{p_{i}^{g} }}$$

Among them.$R_{i}^{g} i \in [1,2,...n]$ is the reliability requirements for $v_{i}$, and $R_{req}^{g}$, the overall reliability of NS and the reliability requirements with each VNF shall meet:

3 $$\prod\limits_{{v_{i} \in V_{g} }} {R_{i}^{g} \ge } R_{req}^{g}$$

In order to ensure the reliability of the 5G- LEO constellation network slice^16^, maximize the deployment benefits of VNF and minimize the overhead of broadband resources, resource migration can be regarded as a way to improve the reliability of the service chain and reduce the mapping cost. According to the VNF reliability weight index, the overall reliability of the 5G- LEO constellation network slice is ensured by meeting the reliability requirements of VNF. Among them,$C1$ is a binary variable constraint. When VNF node $v_{i}$ is mapping to physical node $n$,$x_{{v_{i} ,n}}$ is 1, otherwise is 0; if NSR virtual link $e_{i}$ maps to physical link $l_{nm}$, then $y_{{e_{i} ,l_{nm} }}$ is 1, otherwise it is 0.$C2$ indicates that VNF nodes belonging to the same 5G low orbit constellation network slice cannot be mapped to the same physical node.$C3$ Represents that the reliability $R_{n}^{{}}$ probability value of the physical node is not greater than 1.$C4$ indicates that each VNF has different reliability requirements, and a single VNF is required to meet the reliability requirements in the mapping process.$C5$ while in the physical network without backup protection, the actual overall reliability of 5G low orbit constellation network slice must at least meet the overall reliability requirements of 5G low orbit constellation network slice.$C6$ indicates that the total computing resources required by all VNF nodes in each 5G low orbit constellation network slice deployed to the same physical node are less than or equal to the computing resource capacity of the physical node.$C7$ indicates that the remaining computing resources of the deployed VNF node must be greater than the computing resources required by the node.$C8$ indicates that the sum of bandwidth resources required by all links of each 5G low orbit constellation network slice deployed to the same physical link is less than or equal to the link resource capacity of the physical link.$C9$ indicates that the remaining bandwidth resources of the mapped physical link must be greater than the bandwidth resources required by the virtual link. To solve the above problems, 5G low orbit constellation network slicing requests are divided into three categories: $C_{i}^{g} > C_{n} ,\forall n \in N_{S} ,v_{i}^{g} \in V_{g} ,C_{n} \ge C_{i}^{g}$ ; $R_{n}^{{}} \ge R_{i}^{g} ,n \in N_{S} ,v_{i}^{g} \in V_{g} andC_{n} \ge C_{i}^{g}$;$R_{i}^{g} > R_{n}^{{}} ,\forall n \in N_{S} ,v_{i}^{g} \in V_{g}$, construct 5G low orbit constellation network slice reliability mapping model, and use formula (4) to describe:

4 $$\left\{ \begin{gathered} \mathop {\max }\limits_{{x_{{v_{i} ,n}} ,y_{{ei,l_{nm} }} }} w_{r} \sum\limits_{{g \in N_{S} }} {\sum\limits_{{v_{i}^{{}} \in V_{g} }} {x_{{v_{i} ,n}} C_{i}^{g} \le C_{n} } } - w_{c} (\sum\limits_{{l_{nm} \in L_{s} }} {\sum\limits_{{e_{i} \in E_{g} }} {y_{{ei,l_{nm} }} b_{ei}^{g} } } ) \hfill \\ C1:x_{{v_{i} ,n}} \in \left\{ {0,1} \right\},y_{{ei,l_{nm} }} \in \left\{ {0,1} \right\} \hfill \\ C2:\sum\limits_{{n \in P(V_{g} )}} {x_{{v_{i} ,n}} = 1,\forall v_{i} \in V_{g} } \hfill \\ C3:0 < R_{n}^{{}} < 1,\forall n \in N_{S} \hfill \\ C4:R_{n}^{{}} > R_{i}^{g} ,\forall n \in P^{g} (v_{i} ) \hfill \\ C5:\prod\limits_{{n \in N_{S} }} {x_{{v_{i} ,n}} R_{n}^{{}} \ge } R_{req}^{g} ,\forall v_{i} \in V_{g} \hfill \\ C6:\sum\limits_{{g \in N_{S} }} {\sum\limits_{{v_{i}^{{}} \in V_{g} }} {x_{{v_{i} ,n}} C_{i}^{g} \le C_{n} } } \hfill \\ C7:C_{n}^{r} \ge C_{i}^{g} ,n \in P^{g} (v_{i} ),v_{i} \in V_{g} \hfill \\ C8:\sum\limits_{{g \in N_{S} }} {\sum\limits_{{e_{i} \in E_{g} }} {y_{{ei,l_{nm} }} b_{ei}^{g} \le b_{nm} } } \hfill \\ C9:b_{nm}^{r} \ge b(ei),l_{nm} \in P^{g} (e_{i} ),e_{i} \in E_{g} \hfill \\ \end{gathered} \right.$$

### DQN based 5G low orbit constellation network slice reliability mapping

The 5G low orbit constellation network slice reliability mapping model based on Section "5G low orbit constellation network slice reliability mapping model", taking into account the formula (4) 5G low orbit constellation network slice reliability mapping model parameter set $o$ contain continuous variables such as physical host resource utilization, and the large number of physical host nodes in the production environment will lead to the explosion of 5G low orbit constellation network state space, resulting in reduced reliability of network slice mapping. Therefore, based on the certain decision-making ability of deep reinforcement learning^17^, a reliability mapping method of 5G low orbit constellation network slice based on DQN (deep reinforcement learning) is proposed, and a deep reinforcement network is constructed to fit the 5G low orbit constellation network state behavior value function ^18^, to solve the state space explosion problem in the reliability mapping process of 5G low orbit constellation network slice. This algorithm constructs a neural network with a weight of $\theta$, such that $Q(o,a,\theta ) \approx Q(o,a)^{*}$, of which,$o$ is the parameter set of reliability mapping model for 5G low orbit constellation network slice, that is, the relevant parameters of formula (4),$a$ is action to perform reliability mapping for. In this network, the observation state (the parameter of the reliability mapping model in Formula (4)) is taken as the input, and the two-layer convolutional network is used to process the input, which is used to understand the deployment state of the physical host in the current network, and then the processing results are imported into a full connection layer with ReLU as the activation function. Actual cumulative rewards for using this network $r + \gamma \max_{a^{\prime}} Q(o^{\prime},a^{\prime})$ as the target value.$r$ denotes the immediate reward available to the core controller after performing the mapping action;$\gamma$ indicates the discount factor, which is used to measure immediate and future rewards. The expected cumulative reward $Q(o,a)$ is used as the predicted value, the training purpose is to make the predicted value as close as possible to the target value, so the loss function is defined as.

5 $$L(\theta ) = [r_{l} + \gamma \max_{al^{\prime}} Q(o_{l^{\prime}} ,a_{l^{\prime}} ;\theta ) - Q(o_{l} ,a_{l} ;\theta )]^{2}$$

Compute the partial derivatives of the loss function with respect to the weights of the neural network:

6 $$\frac{\partial L(\theta )}{{\partial \theta }} = [r_{l} + \gamma \max_{al^{\prime}} Q(o_{l^{\prime}} ,a_{l^{\prime}} ;\theta ) - Q(o_{l} ,a_{l} ;\theta )] \times \frac{{\partial Q(o_{l} ,a_{l} ;\theta )}}{\partial \theta }$$

**Figure 3:**
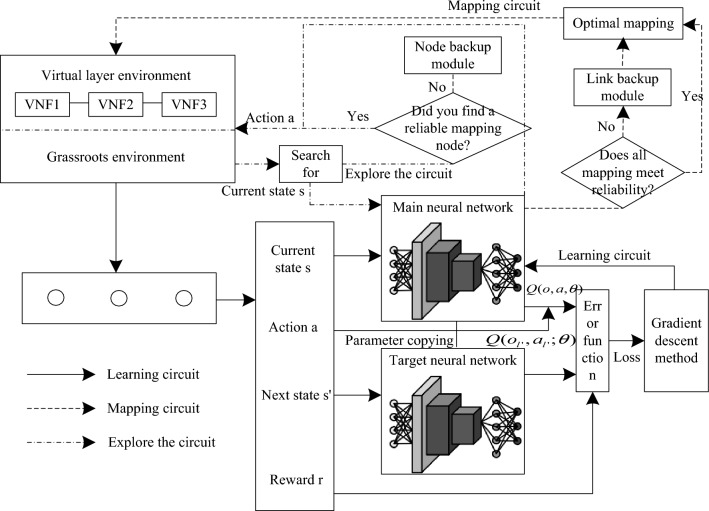
Schematic diagram of 5G low orbit constellation network slice mapping based on DQN.

By using gradient descent method and back propagation mechanism for several iterations, it is possible to find $Q(o,a,\theta )$. In the neural network training process, the training samples used need to have the property of being independently and identically distributed, and the two neighboring training samples in the above process $\left\langle {o_{l} ,a_{l} } \right\rangle$ and $\left\langle {o_{l^{\prime}} ,a_{l^{\prime}} } \right\rangle$ are correlated, the neural network will overfit the training process and the learned experience cannot be generalized. Therefore, an experience replay cache pool and a target network are introduced into the network to break the correlation. Where the experience replay cache pool is used to record migration information, the $\left\langle {o_{l} ,a_{l} ,o_{l^{\prime}} ,a_{l^{\prime}} } \right\rangle$, while randomly sampling from the playback cache pool to obtain migration information $\left\langle {o_{j} ,a_{j} ,o_{j^{\prime}} ,a_{j^{\prime}} } \right\rangle$ as the real training input to the neural network. The target network is a network with weights as $\theta ^{\prime}$, every after a number of training to update the target network, so that the target value is relatively fixed, so as to prevent the training process overfitting. The flow of the algorithm is shown in Fig. 3.

### Code availability

The code for studying the reliability mapping of 5G low orbit constellation network slices should be modularized for easy understanding, modification, and expansion. The implementation of gradient descent and backpropagation mechanism should have clear interface definitions for each module to interact with other modules. Pseudocode under gradient descent and backpropagation mechanism:

- Input: min (float X ,float Y)
- { int Z;
- Z = X > Y?X:Y
- return (Z);
- Ouput: The results of SDE.
- end}

Using deep reinforcement learning to select the path for generating reliable mapping codes for 5G low orbit constellation network slicing, node segmentation is performed on all required information generated by embedded software code generation. According to the requirements of automatic code generation, the path traversal depth is reasonably set. The more native code information, the greater the coverage depth of the selected path, and the more paths can be selected for automatic code generation.

The formula for calculating path coverage depth is:

7 $$S_{D} = \frac{{\sum\limits_{i = 1}^{n} {(U_{i} - \mu_{i} )} }}{n}$$

**Figure 4:**
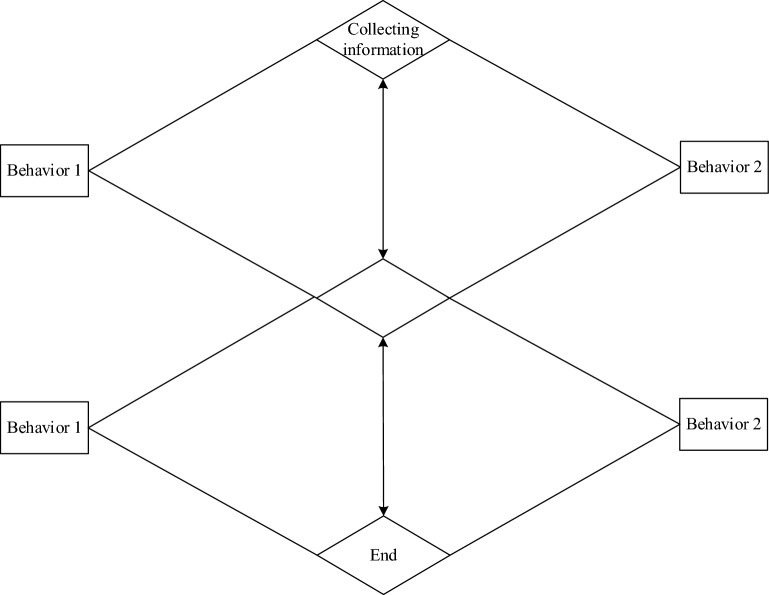
Path Generation Process.

In the formula, $U_{i}$ represents the similarity between code nodes; $\mu_{i}$ represents the level of assessed risk obtained; $n$ represents the number of evaluated nodes. The path generation process is shown in Fig. 4.

According to Fig. 4, the 5G Low Orbit Constellation Network Slice Reliability Mapping System randomly allocates the code information to be generated into multiple test path sets, arranges the code generation paths of concurrent structures in embedded software and the structural paths within branches in order, outputs effective code generation paths, and selects the best 5G Low Orbit Constellation Network Slice Reliability Mapping code generation path based on the shortest path as the selection principle within the generated paths, achieving code availability research.

### Backup programs that consider reliability

The deployment plan in Sect. "Dqn based 5g low orbit constellation network slice reliability mapping" and the availability of 2.5 code can obtain reliable 5G low orbit constellation network slice mapping results. However, there may be no physical nodes that meet the VNF reliability requirements within the mapping range during VNF deployment. In addition, the link reliable mapping model appropriately improves the link mappable length, which requires link backup to meet the reliability. Therefore, in order to improve the mapping success rate, corresponding backup schemes are required.

#### Node backup program

The node backup scheme is an important measure to ensure the reliability of the network slice in the case of failure or congestion. The specific content of this backup scheme involves configuring redundant nodes or resources in the network so that the main node can take over its work quickly when there is a problem. In sharing mode, backup nodes or resources can be shared by multiple network slices to improve resource utilization and cost-effectiveness. Multiple LEO constellation network slices can share a set of backup satellites or ground stations to provide backup services when needed. By collecting network state information and training the model, the algorithm can learn how to dynamically adjust the configuration and use of backup resources according to network conditions and business requirements.

Filtering the action space, including path length constraints, resource constraints, and reliability constraints, gets the set is:

8 $$\begin{gathered} N^{\prime}_{{kn_{i} }} = \left\{ {n_{i} \left| {hop(s_{k} ,n_{i - 1} ) + hop(n_{i - 1} ,n_{i} ) + hop(n_{i} ,d_{k} ) \le u^{\prime}_{k} ,R_{{n_{i} }} \ge (R_{N}^{k} )^{\omega I} ,c_{nI}^{k} < c_{i} } \right.} \right\} \cdot p_{{sk,n_{i - 1} }} \in p_{{sk,v_{I - 1}^{k} }} ,p_{{n_{i - 1} ,n_{i} }} \hfill \\ \mathop {}\nolimits_{{}}^{{}} \mathop {}\nolimits_{{}}^{{}} = l_{{n_{i - 1} ,n_{i} }} ,p_{{n_{i} ,d_{k} }} = l_{{n_{i} ,d_{k} }} ,n_{i} \in h(v_{I}^{k} ) \hfill \\ \end{gathered}$$

Among them,$s_{k}$ is the network data flow entry point.$d_{k}$ is the location where the data stream is delivered; the maximum value of transmission hops is $u^{\prime}_{k}$; The reliability requirements for $v_{I}^{k}$ is $(R_{N}^{k} )^{\omega I}$; $c_{nI}^{k}$ is the computational resource requirements for $v_{I}^{k}$. When the mapped nodes are determined, the set $L^{\prime}_{{kl_{i,j} }}$ of mapped links can be determined, from which the minimum path $hop(i,j)b_{I,J}^{k} /b_{i,j}$ is chosen, making their actions more conducive to achieving the optimization goal.

When VNF reliability requirements is high, when $N^{\prime}_{{kn_{i} }}$ is empty, based on the idea of backup, two deployment nodes can be selected at the same time, one of which is the backup node. At this time, the node set is:

9 $$\begin{gathered} N^{\prime\prime}_{{kn_{i} }} = \left\{ {n_{i} \left| {hop(s_{k} ,n_{i - 1} ) + hop(n_{i - 1} ,n_{i} ) + hop(n_{i} ,d_{k} ) \le u^{\prime}_{k} ,c_{nI}^{k} < c_{i} } \right.} \right\} \hfill \\ \mathop {}\nolimits_{{}}^{{}} \mathop {}\nolimits_{{}}^{{}} p_{{s_{k} ,n_{i - 1} }} \in p_{{s_{k} ,v_{I - 1}^{k} }} ,p_{{n_{i - 1} ,n_{i} }} = l_{{n_{i - 1} ,n_{i} }} ,p_{{n_{i} ,d_{k} }} = l_{{n_{i} ,d_{k} }} ,n_{i} \in h(v_{I}^{k} ) \hfill \\ \end{gathered}$$

In order to effectively improve the reliability of 5G low orbit constellation network slice mapping^19^, and improve the utilization rate of backup resources, the backup mode is set for the importance of VNF. By normalizing the importance, a dedicated backup is performed for those higher than the average SFC importance. The physical nodes with higher reliability are used for deployment, and the smaller ones are used for backup. On the contrary, shared backup can further reduce resource consumption while ensuring that important VNFs do not fail. At this time, select the deployment node in $N^{\prime\prime}_{{kn_{i} }}$, and the backup node is used as the node mapping action.

#### Link backup algorithm

Since the deployment result may not meet the reliability of the 5G low orbit constellation network virtual link^20,21^, link backup is required at this time. For link delay, besides normal distribution, other distributions that can better capture the actual network conditions need to be considered, such as lognormal distribution, exponential distribution or Weibull distribution. These distributions can provide a more accurate simulation of delay behavior. For network traffic generation, Poisson process cannot fully reflect the complex and diverse traffic patterns in 5G networks. Consider using a hybrid model to simulate network traffic, and combine different stochastic processes to capture the behaviors of different types of traffic. Using network simulation tools is a powerful means to simulate and analyze the performance of 5G networks. These tools allow people to configure various parameters and scenarios, and generate detailed simulation results, so that people can better understand and optimize network performance. In order to select an appropriate path to be backed up, and to achieve sufficient reliability increment with minimum link resource consumption as the goal, the reliability improvement rate of unit resource is proposed:

10 $$\sigma_{I,J}^{k} = \Delta /BW$$

11 $$\Delta = \prod\limits_{{l_{i,j} = g(l_{I,J}^{k} ),l_{I,J}^{k} \in L_{v}^{k} }} {RL(l_{i,j} )} \cdot (1 - \prod\limits_{{l_{{_{i,j} }}^{*} = g(l_{I,J}^{*k} ),l_{I,J}^{*k} \in L_{v}^{k} }} {RL(l_{i,j} )} )$$

$BW$ is the consumption of resources for link backup $l_{i,j}$.$l_{{_{i,j} }}^{*}$*is the deployed links. In order to improve the reliability of low reliability virtual links, then:

12 $$\chi_{I,J}^{k} = \sigma_{I,J}^{k} /\prod\limits_{{l_{i,j} \in g(l_{I,J}^{k} )}} {RL(l_{i,j} )}$$

$\chi_{I,J}^{k}$ effectively evaluates the importance of link $l_{I,J}^{k}$ in the backup process to achieve less costly link resources and greater reliability improvement, sort $l_{I,J}^{k}$ by $\chi_{I,J}^{k}$, from large to small iterative backup to meet the reliability requirements.

## Experimental analysis

In order to verify the effectiveness of the 5G low orbit constellation network slice reliability mapping of the method in this paper, the experimental environment of repeatable experiments is constructed as shown in Fig. 1, and the cloud computing simulation platform CloudSimSDN is expanded to realize the business flow generation module and algorithm test module. CloudSimSDN is a cloud computing simulator used to build the underlying network environment, including simulating network topology and physical host resources, monitoring system operation, and analyzing system energy consumption. The network topology contains 45 nodes, which are connected in a tree form, and the link delay is randomly generated according to the normal distribution. The network flow generation module is used to generate network traffic based on Poisson process. In this study, it is agreed that the length of SFC is 10–30, and there are no more than 10 types of VNF. The algorithm test module realizes the validation of the reliability mapping of 5G low orbit constellation network slice by the method in this paper, and uses Java language to write code. The experimental hardware environment is Lenovo T480S computer, equipped with Intel Core i7-8550U 8 core processor, GeForce MX150 2 GB GPU, DDR416GB memory, and the operating system is Ubuntu 18.04Server. Relevant parameters are shown in Table 1.

**Table 1:** Experimental Parameters.

Experimental parameters	Numerical value
Number of physical nodes	45
Slice request reliability threshold	[0.93, 0.99]
Physical node reliability	[0.95,0.99]
Physical node CPU resource capacity	[10.20]
Bandwidth capacity between physical nodes	[30,60]
VNF computing resource requirements	[3, 9]
Bandwidth requirements between VNFs	[8, 16]
The number of VNFs requested for slicing	[4, 8]
Iterations	500
Playback buffer pool capacity	100
Resource overbooking threshold	0.3
Exploring Development Decision Thresholds	0.9
Transmission delay weight	0.2
Queuing delay weight	0.8
Reward balance constant	60

In order to verify the reliability mapping effect of 5G low orbit constellation network slice in this method, corresponding simulation failure scenarios are designed for three situations: network equipment failure, satellite communication failure and network congestion. The failure recovery time, recovery success rate, throughput, packet loss rate and intra slice traffic are taken as evaluation indicators. Before fault scenario simulation, measure and record the throughput, packet loss rate and intra slice traffic of 5G low orbit constellation network slice mapping, and use them as the baseline. In the simulated fault scenario, monitor the performance of 5G low orbit constellation network slice mapping in real time, and record the changes of these real-time data; After the end of the simulated fault scenario, observe and record the fault recovery time and recovery success rate of 5G low orbit constellation network slice mapping, and record the repaired throughput, packet loss rate and intra slice traffic. By comparing and analyzing the real-time monitoring and recorded data with the previous baseline data, observe the performance changes of 5G low orbit constellation network slice mapping under the three fault scenarios. The verification results are shown in Table 2.

**Table 2:** Mapping Results of 5G Low Earth Orbit Constellation Network Slices Using the Method in this paper.

Fault Type	No fault	Network device failure	Satellite communication failure	Network congestion	After repair
Throughput/kbps	13	10	11	8	15
Packet loss rate/%	1	6	12	8	0
Slice flow rate/piece	35	26	19	21	37
Fault recovery time/s	–	0.29	0.12	0.25	–
Recovery success rate/%	–	100	100	100	–

Table 2 clearly depicts the changes in performance indices of the 5G low orbit constellation network under various fault conditions. In scenarios where network equipment fails, satellite communication encounters issues, or the network becomes congested, the transmission efficiency and reliability of the network are significantly impacted, resulting in a noticeable downward trend in network throughput, packet loss rate, and intra-slice traffic. However, after implementing the processing method proposed in this paper, the performance of the 5G low orbit constellation network has been considerably enhanced. In terms of throughput, packet loss rate, and intra-slice traffic, the processed network exhibits superior performance. Notably, even in the event of network failures, these three indicators have improved compared to their pre-failure state. Furthermore, the failure recovery time of the 5G low orbit constellation network processed by this method is less than 0.3 s, indicating its ability to restore normal operation promptly in the event of a failure. The recovery success rate achieves 100%, further validating the effectiveness of this method in enhancing network reliability.

**Figure 5:**
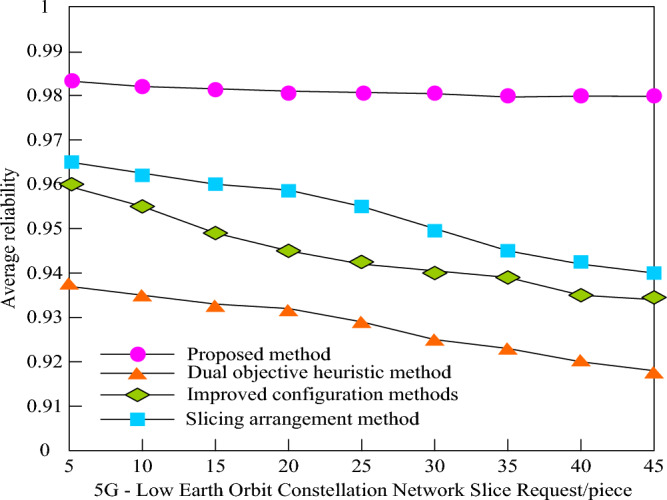
Reliability of 5G Low Earth Orbit Constellation Network Slice Mapping with Different Methods.

In order to verify the reliability mapping capability of 5G low orbit constellation network slices of this method, set the number of 5G low orbit constellation network slices at 5–45, and calculate the average reliability of the double objective heuristic method, improved configuration method, network slice arrangement method and this method. Figure 5 shows the average reliability change of the four methods when processing 5G low orbit constellation network slice requests.

As illustrated in Fig. 5, as the number of 5G low orbit constellation network slice requests rises, the network load increases, causing an augmentation in the failure rate of service processing and data transmission. Consequently, the average reliability of the four methods exhibits a downward trend. When the number of 5G low orbit constellation network slicing requests is 5, the dual objective heuristic method achieves an average reliability of 93.8%, the improved configuration method achieves 96%, the slicing arrangement method achieves 96.5%, and the method presented in this paper achieves 98.4%. This indicates that, with a small number of requests, the method proposed in this paper is more reliable than the other three methods. As the number of requests for 5G low orbit constellation network slicing increases to 45, the reliability of the dual objective heuristic method, the improved configuration method, and the slicing arrangement method decreases by approximately 2%. However, the reliability of the method presented in this paper remains above 98%. In comparison to the first three methods, the method proposed in this paper maintains high reliability even when facing a large number of requests.

**Figure 6:**
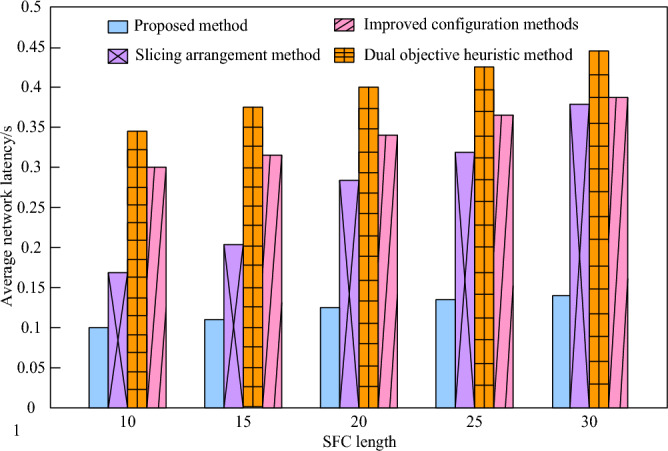
Average network latency of different methods.

In order to further verify the effectiveness of the 5G low orbit constellation network slice reliability mapping of the method in this paper, compare the average network delay of the four methods when the service function chain (SFC) length range is 10–30 with the two objective heuristic method, the improved configuration method, the network slice layout method and the method in this paper. The comparison results are shown in Fig. 6.

As evident from Fig. 6, in the 5G low orbit constellation network slicing environment, as the length of the service function chain (SFC) increases, encompassing more network components and services, the processing and communication time also elongates, leading to a corresponding rise in the average delay of the four methods. For a given SFC length, the double objective heuristic method generates the highest average delay, indicating its inefficiency in handling SFCs and significant performance limitations in realizing the service function chain. While the improved configuration method shows some improvement compared to the double objective heuristic method, its average delay remains high and does not meet the desired performance levels. When the SFC length is 10, the network slicing method exhibits a low delay, but as the SFC length increases, its delay growth rate is the fastest, indicating that it performs better with shorter SFCs but declines more rapidly when dealing with longer ones. In contrast, the method proposed in this paper exhibits a slow delay growth rate, consistently remaining below 0.15 s. This demonstrates the good stability and performance of the method when handling SFCs of varying lengths.

**Figure 7:**
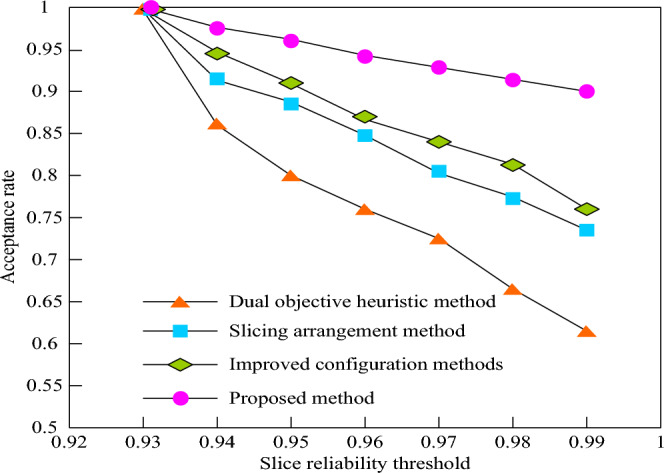
Request acceptance rates for different methods.

Request acceptance rate is an important method to evaluate the reliability of 5G low orbit constellation network slice mapping, which helps to understand the performance and stability of the system, and provides a basis for further optimization and improvement. The request acceptance rate is the ratio of the number of successfully mapped slice requests to the total number of arriving requests under the reliability threshold constraint. A higher reliability threshold means that more physical resources are needed to ensure the reliability of the service. In order to verify the reliability of 5G low orbit constellation network slice mapping in this method, in this simulation experiment, set the total number of requests to 20. When the slice reliability threshold is 0.93–0.99, compare the request acceptance rate of the dual objective heuristic method, improved configuration method, network slice orchestration method and this method, so as to evaluate the performance of the four methods in providing reliable services. Figure 7 shows how the request acceptance rate of the four methods changes with the slice reliability threshold in the experimental simulation.

It is evident from Fig. 7 that, with a fixed amount of physical resources, an increase in the reliability threshold leads to resource insufficiency, resulting in a downward trend in the acceptance rate percentage of 5G low orbit constellation network slice service requests. As the reliability threshold rises, to meet higher reliability demands, more redundant resources are required to handle potential service failures. This increased allocation of redundant resources reduces the availability of resources, placing greater pressure on the redundancy allocation of virtual network functions, which further decreases the request acceptance rate. The low acceptance rate observed in the double objective heuristic method suggests that the method is inefficient in resource allocation and redundancy management, resulting in a shortage of available resources. In comparison, the improved configuration method and slicing arrangement method show some improvement, but when the reliability threshold rises to 0.99, the slicing request acceptance rate drops to approximately 75%. However, the method presented in this paper achieves the highest acceptance rate percentage in all scenarios, surpassing 90%. This demonstrates that this method excels in both resource allocation and redundancy management, making more effective use of limited physical resources and enhancing service reliability.

**Figure 8:**
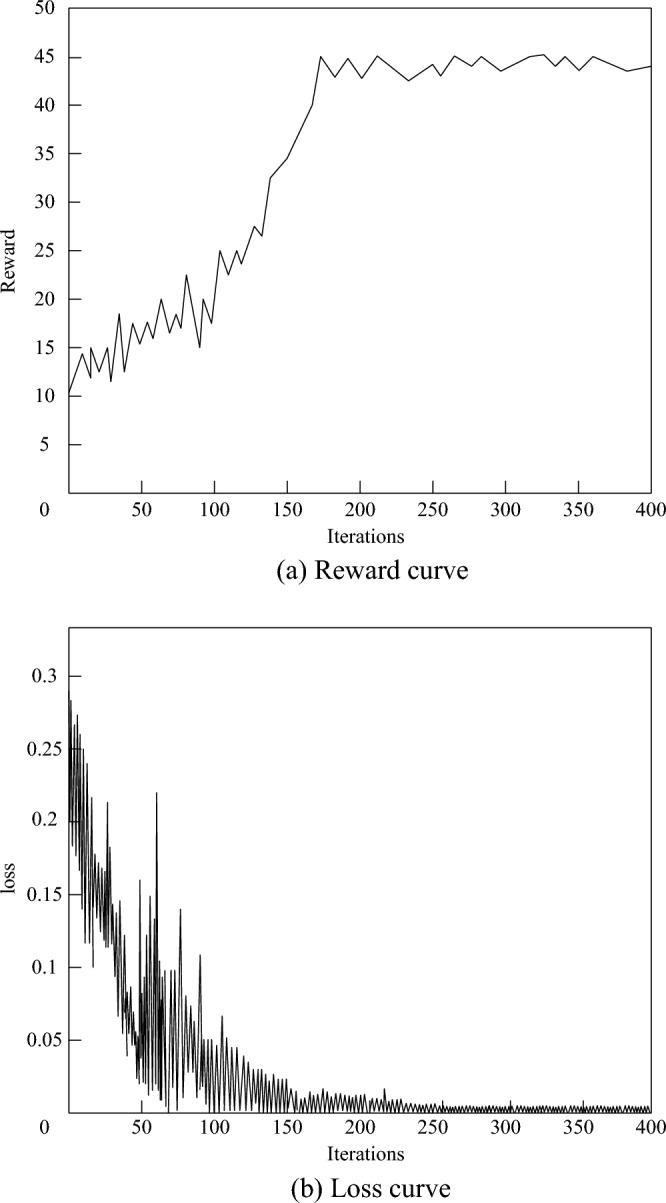
Convergence effect of the method proposed in this paper.

As an important indicator to measure the performance of this method, the reward curve can intuitively display the change of the reward value of this method in the training process. By observing the trend of the reward curve, we can understand whether this method can gradually improve the performance in the training process and eventually become stable. If the reward curve shows a gradually increasing trend, And it reaches a stable state in the late training period, which will strongly prove the effectiveness of the method in this paper. At the same time, the loss curve is also an important basis for evaluating the performance of the method in this paper. The loss value directly reflects the loss of the algorithm in the training process, that is, the gap between the current strategy and the optimal strategy. With the training, the loss value should gradually decrease and eventually become stable. By analyzing the change trend of loss curve, the convergence effect of this method on the reliability mapping problem of 5G low orbit constellation network slice can be further verified. The verification results are shown in Fig. 8.

Figure 8a depicts the reward curve of the method presented in this paper. As the number of training iterations gradually increases, it is evident that the reward value obtained after each iteration exhibits a steadily increasing trend. Once the number of training iterations reaches approximately 170, the reward value begins to stabilize, suggesting that the method has gradually converged to a relatively stable strategy. However, during this process, a punishment mechanism is occasionally triggered, leading to a sudden dip in the reward value. The purpose of this punishment mechanism is to encourage the algorithm to further explore and optimize the strategy, thus preventing it from getting stuck in a local optimum. Figure 8b illustrates the loss curve of the method. As the number of training iterations increases, it is noticeable that the loss function gradually decreases. In the initial training stages, the loss function decreases significantly, indicating rapid learning and optimization of the strategy. However, as training progresses, the decrease in the loss function becomes more gradual, ultimately reaching a relatively stable state after 150 to 250 training iterations. This indicates that the neural network has gradually converged, thus verifying the effectiveness of this method in addressing the reliability mapping issue of 5G low orbit constellation network slices.

## Conclusion

The traditional network slice mapping method ignores the different reliability requirements of VNF, which leads to poor reliability of the slice. In order to ensure the reliability requirements of VNF, based on the 5G low orbit constellation network scenario, this paper studies the 5G low orbit constellation network slice reliability mapping method based on deep reinforcement learning. The experimental results prove that this method ensures the throughput, traffic transmission delay and data packet loss rate of 5G low orbit constellation network, improves the load balance of 5G low orbit constellation network, and thus improves the reliability of the network; Compared with other methods, this method has higher reliability with the same number of requests; This method uses node reliability importance mapping to carry out resource allocation and redundancy management, which can better identify key nodes and allocate more resources to them, thus improving the reliability and availability of network services.

In a larger network scenario, the state space that agents need to deal with (that is, possible network configuration, traffic patterns, etc.) will increase significantly, and the deep reinforcement learning algorithm needs to have the ability to deal with this large-scale state space to ensure that effective strategies can be found. With the increase of network scale, the computational requirements of deep reinforcement learning algorithm will increase accordingly, including the computational resources needed for training neural networks and the resources needed for implementing strategies in the actual environment. In order to meet these requirements, high-performance computing resources, such as GPU cluster or cloud computing platform, need to be adopted. In addition, the design of the algorithm also needs to consider how to effectively use these resources to achieve better scalability.

### Ethical approval

The research met all applicable standards for the ethics of experimentation.

## Data Availability

The datasets generated during and/or analyzed during the current study are available from the corresponding author on reasonable request.

## References

[1] Valente F, Eramo V, Lavacca FG (2023). Optimal bandwidth and computing resource allocation in low earth orbit satellite constellation for earth observation applications. Comput. Netw..

[2] Datar M, Altman E, Le Cadre H (2023). Strategic resource pricing and allocation in a 5g network slicing stackelberg game. IEEE Trans. Netw. Serv. Manage..

[3] Soret B, Leyva-Mayorga I, Cioni S, Popovski P (2021). 5g satellite networks for internet of things: offloading and backhauling. Int. J. Satell. Commun. Network..

[4] Tsuchida H, Kawamoto Y, Kato N, Kaneko K, Aruga H (2020). Efficient power control for satellite-borne batteries using q-learning in low-earth-orbit satellite constellations. IEEE Wireless Communications Letters.

[5] Wang W, Chen T, Ding R, Seco-Granados G, Gao X (2021). Location-based timing advance estimation for 5g integrated leo satellite communications. IEEE Trans. Veh. Technol..

[6] Yu H, Musumeci F, Zhang J, Tornatore M, Ji Y (2020). Isolation-aware 5g ran slice mapping over wdm metro-aggregation networks. J. Lightwave Technol..

[7] Thiruvasagam PK, Kotagi VJ, Murthy CSR (2020). A reliability-aware, delay guaranteed, and resource efficient placement of service function chains in softwarized 5g networks. IEEE Trans. Cloud Comput..

[8] Zanzi L, Sciancalepore V, Garcia-Saavedra A, Schotten HD, Costa-Perez X (2021). Laco: a latency-driven network slicing orchestration in beyond-5g networks. IEEE Trans. Wireless Commun..

[9] Manogaran G, Ngangmeni J, Stewart J, Rawat DB, Nguyen TN (2023). Deep-learning-based concurrent resource allocation method for improving the service response of 6g network-in-box users in uav. IEEE Internet Things J..

[10] Shabka Z, Zervas G (2023). Network-aware compute and memory allocation in optically composable data centers with deep reinforcement learning and graph neural networks. J. Opt. Commun. Netw..

[11] Lin Z, Lin Z, Huang Z (2021). Low latency and high-reliability data query algorithm in deep double Q network. Comput. Simul..

[12] Lee Y, Choi JP (2021). Connectivity analysis of mega-constellation satellite networks with optical intersatellite links. IEEE Trans. Aerosp. Electr. Syst..

[13] Mcdonell T (2021). Orchestrating a new multi-orbit broadband constellation. Space News Int..

[14] Goncalves DM, Puliafito C, Mingozzi E, Bittencourt LF, Madeira ERM (2023). End-to-end network slicing in vehicular clouds using the mobfogsim simulator. Ad hoc Netw..

[15] Saibharath S, Hota C, Mishra S (2023). Joint qos and energy-efficient resource allocation and scheduling in 5g network slicing. Comput. Commun..

[16] Rajagopal A, Ramachandran A, Shankar K, Khari M, Joshi GP (2020). Optimal routing strategy based on extreme learning machine with beetle antennae search algorithm for low earth orbit satellite communication networks. Int. J. Satell. Commun. Netw..

[17] Demizu T, Fukazawa Y, Morita H (2023). Inventory management of new products in retailers using model-based deep reinforcement learning. Expert Syst. Appl..

[18] Paul A, Mitra S (2022). Deep reinforcement learning based cooperative control of traffic signal for multi-intersection network in intelligent transportation system using edge computing. Trans. Emerg. Telecommun. Technol..

[19] Ahmad A, Kumar N (2023). Knowledge-based flexible resource allocation optimisation strategy for multi-tenant radio access network slicing in 5g and b5g. Int. J. Ad Hoc Ubiquitous Comput..

[20] Chen W, Smieliauskas W, Liu S (2020). Study on the reliability assessment and early-warning method of online auditing based on the perspective of it control. Grey Syst. Theory Appl..

[21] Liu YC, Zhang JN (2024). Service function chain embedding meets machine learning: Deep reinforcement learning approach. IEEE Trans. Netw. Serv. Manag..

